# Behavioural and neural modulation of *win-stay* but not *lose-shift* strategies as a function of outcome value in Rock, Paper, Scissors

**DOI:** 10.1038/srep33809

**Published:** 2016-09-23

**Authors:** Lewis Forder, Benjamin James Dyson

**Affiliations:** 1 University of Sussex, Falmer, BN1 9QH, UK

## Abstract

Competitive environments in which individuals compete for mutually-exclusive outcomes require rational decision making in order to maximize gains but often result in poor quality heuristics. Reasons for the greater reliance on *lose-shift* relative to *win-stay* behaviour shown in previous studies were explored using the game of Rock, Paper, Scissors and by manipulating the value of winning and losing. Decision-making following a loss was characterized as relatively fast and relatively inflexible both in terms of the failure to modulate the magnitude of *lose-shift* strategy and the lack of significant neural modulation. In contrast, decision-making following a win was characterized as relatively slow and relatively flexible both in terms of a behavioural increase in the magnitude of *win-stay* strategy and a neural modulation of feedback-related negativity (FRN) and stimulus-preceding negativity (SPN) following outcome value modulation. The *win-stay*/*lose-shift* heuristic appears not to be a unified mechanism, with the former relying on System 2 processes and the latter relying on System 1 processes. Our ability to play rationally appears more likely when the outcome is positive and when the value of wins are low, highlighting how vulnerable we can be when trying to succeed during competition.

There are a number of serious and playful aspects of society in which individuals recursively engage with others for mutually-exclusive outcomes in a non-cooperative fashion^1^: there will be only one job awarded to the pool of applicants, there will be only one winner at poker. Success in these environments calls for rational decision-making and sound strategic planning (e.g., System 2) but due to the impact of emotion and arousal in these contexts^2^, performance is often compromised by reliance on more intuitive and impulsive action (e.g., System 1; ref. 3). Individuals who exhibit domain mastery appear to have subdued some of the tensions between System 1 and System 2: professional poker players regulate their emotional response to losses (ref. 4; i.e., reliance on System 2 rather than System 1), whereas for board game experts, their intuitive response tends also to be their best response (ref. 5; i.e., refinement of System 1 output). For the rest of us, these environments all-too-clearly betray the bounded rationality of human decision-making^6^. The game of Rock, Paper, Scissors (RPS) serves as one such context to study the tension between System 1 and System 2 activity, acting as a relatively simple game space but yielding complex and cyclical patterns of behaviour^7^.

The only guaranteed safe way to play RPS is to adopt the mixed equilibrium strategy (c.f., ‘*minimax* solution’; ref. 8) wherein one plays randomly with respect to the previous event^9^ while also ensuring that all three responses are played 33% of the time across the entire number of trials^10^. If both players adopt this strategy, then this unique Nash equilibrium leads to a zero-sum game^11^ where neither player experiences significant gains in the long run, but also ensures that neither player can be dominated by their opponent. Unfortunately, such processes are likely to be outside the bounds of human cognition, and instead individuals tend to employ heuristics during gameplay that run the risk of being dominated by opponents.

One common heuristic in RPS is *win-stay lose-shift*^7^,^12^,^13^. Such tendencies have their roots in behaviourism (c.f., ref. 14) where responses associated with reinforcement are more likely to be repeated and responses that are associated with punishment are more likely to be changed. Such principles may be fundamental properties of any successfully learning organism but in the context of a competitive environment, the predictability of repeating a response following a positive outcome and changing a response following a negative outcome is evolutionarily unsound. For example, an opponent savvy to a player overusing the *win-stay* strategy could begin to make dominating counter-moves so if, for example, a player won on Rock on the first trial, their opponent would be likely to play Paper on the second trial. In a previous paper^7^ we showed that deviations from rational (*minimax*) decision making in RPS were more likely following negative (e.g., *lose* and *draw*) rather than positive (e.g., *win*) trials (c.f., ‘tilting’ in poker; ref. 4). This was shown by the rough equivalence of the proportion response to each of the three available strategies: one *stay* response and two switch responses (*upgrade*, *downgrade*) following a *win* trial, but the predominance of switching strategies following negative outcome (specifically, *downgrading* [selecting the item that would have been beaten by the player’s previous response] following *loss* and *upgrading* [selecting the item that would have beaten the player’s previous response] following *draw*^15^). A similar reluctance to adopt *win-stay* relative to *lose-shift* behaviour has also been noted in human reversal learning^16^ and also spatial discrimination learning in rats^17^ (although see the primate work of^12^ for a contrary position). From an evolutionary point of view, failure to initiate behavioural change following a *loss* (or even the perceived threat of *loss*) is likely to be more damaging than the failure to repeat an action following a *win*. To wit: “Neither the mouse nor the gazelle can afford to *learn* to avoid”^18^ (p. 33, emphasis in original).

This paper focuses on why negative outcomes should be more likely to lead to irrational decision-making, and whether performance based on outcome-strategy contingencies can be modulated and possibly corrected. One reason why losses should have a more pronounced effect on behaviour is that the subjective value of wins and losses are not the same as their objective value. Specifically, losses are judged to be roughly twice as bad as gains^19^. In our previous RPS study^7^, we did not use a point or monetary system but we might assume that objectively a win and a loss trial were ‘worth’ the same value (i.e., +1 and −1) in the larger context of the game. Therefore, to examine decision making under conditions where wins are assigned +1, losses are assigned −1 and draws are assigned 0, is perhaps to produce an imbalance in the subjective value of loss (e.g., ≈ −2). It may be the larger subjective value of the loss rather than the negative valence of the loss per se that led to predictable deviation from rational decision making. To address this, we introduced a point system during RPS play, comparing performance in a *baseline* condition (where +1 for win, −1 for loss, 0 for draw) against two additional conditions (see Fig. 1a; following^6^). In the *win-heavy* condition, point values of +2, −1 and 0 were assigned to *wins*, *losses* and *draws*. Here, the assigned value of a win (+2) equated the subjective value of a loss (e.g., ≈ −1 * 2). If it is the subjective magnitude of the outcome rather than valence that drives an individual towards irrational decision making, then we would expect an increase in the observation of *win-stay* strategizing relative to *baseline* performance in this condition. In the *lose-heavy* condition, these values were +1, −2 and 0, respectively. By the same logic, in this condition losses have approximately four times the value as wins (e.g., ≈ −2 * 2 for *loss* versus +1 for *win*) so we would expect an exacerbation in *lose-shift* strategizing relative to *baseline* performance.

A second reason why losses might impact on subsequent performance to a greater extent than wins is due to the immediate attentional capture afforded by a losing relative to a winning action. In a variant of a dot-probe task using RPS^20^, participants were asked to observe play trials and then to respond to the quantity of dots displayed either behind the winning response or the losing response. It was found that dot-probe responses were faster when presented in the location of the losing response. We extrapolate these findings to the initiation of decisions *following* losing and winning. Since heuristics are characterised as being ‘fast and frugal’^21^, cases of irrational decision making (frugal) should also be associated with shorter RTs (fast). Therefore, in addition to a baseline tendency of increased irrational play following *lose* trials relative to *win* trials, the time to initiate these former responses should also be faster.

We also augment our behavioural predictions regarding heuristic modulation with the simultaneous recording of neural activity. A well-documented neural signature of outcome sensitivity is feedback-related negativity (FRN or fERN; ref. 22). The FRN is observed roughly 200–300 ms following the on-set of feedback, appears maximal at central and fronto-central electrodes, and originates from anterior cingulate cortex^23^,^24^. The FRN is thought to be distinct from a second type of performance monitoring known as ERN or error-related negativity^22^ (see also the dispute over medial-frontal negativity [MFN] and its potential role in sensitivity to financial outcome^25^,^26^). In the case of ERN, amplitude is larger as a result of *internally* generated failure such as an incorrect motor response, whereas FRN is larger as a result of *externally* generated failure such as losing against an opponent^27^. Both are thought to have their genesis in the release of dopamine in the basal ganglia^28^,^29^, although the relationship between FRN and dopamine also implicates norepinephrine, serotonin, GABA, and adenosine^30^. The FRN is then a potentially critical adaptive response where signals of success and failure ultimately project to a higher-order decision making network, where behaviour can be changed in the light of previous performance^29^.

The paradigm of RPS is well positioned to examine FRN for a number of reasons. First, RPS performance against an opponent adopting the mixed-equilibrium strategy ensures an approximately equal distribution of wins and losses across the series, thereby reducing the overall contribution of outcome expectancy effects^24^,^31^. This is similarly in keeping with the suggestion of^32^ who argue that during examination of FRN, trial outcome should not be controlled by the participant. Second, the possibility of draw trials in RPS, in addition to win and loss trials, satisfies the need in the FRN literature for the examination of neutral trials^29^,^31^. Third, by modulating the values assigned to the three types of trial outcome across *baseline*, *win-heavy*, and *lose-heavy* conditions we are able to adjudicate between competing theories of FRN (see ref. 32): For some researchers^33^, the FRN represents a binary process, distinguishing between positive and negative outcomes only. Evidence for this position is derived from the observation that FRN failed to modulate as a function of the degree of loss experienced by a participant^34^ and that under the examination of positive, negative and neutral feedback, neutral feedback appears similar to negative feedback^29^. Collectively, these observations would appear to conceptualise the FRN as an index of whether a current goal is achieved (e.g., *win*) or not achieved (e.g., *lose* or *draw*). Under the position that the FRN is a binary process responsive to valence, we should expect differences between goal-consistent and goal-inconsistent outcomes, but that these differences should not modulate as a function of *baseline*, *win-heavy* or *lose-heavy* condition. For others, the FRN is sensitive to the magnitude of certain trial outcomes and^35^ provide evidence for FRN modulation relative to the probability of winning across blocks of trials (but not the probability of losses, c.f., the discussion of^29^ above). Under the alternative position that the FRN is sensitive to the magnitude of positive or goal-consistent outcomes, then we should expect neural modulation across *win* trials as a function of the value and context within which the positive outcome is placed.

An alternative interpretation of FRN suggests that it is only generated when feedback represents non-redundant information^32^. This is potentially problematic in the current paradigm since there is full disclosure of response information (e.g., opponent played Paper, player played Rock) prior to a separate feedback display (e.g., *lose*; as in^7^). Hence, an attentive player may be able to infer a ‘loss’ trial upon the presentation of both responses, thereby rendering the explicit presentation of ‘lose’ in a separate feedback display redundant ref. 26 discuss a similar possibility in the context of ERN: “when the stimulus–response mappings are fixed and can be learned, the ERN should occur at the time of the error response and not at the time of feedback presentation…on such trials the system detects the error at the time of the response, producing a negative prediction error and a large ERN, and so by the time of feedback presentation the system has already detected the error, thus producing no change in prediction and no ERN…” (p. 251; see also^36^, in the context of FRN). One way to address this concern would be to examine neural activity prior to the on-set of the feedback display. Therefore, as a second metric of neural modulation, we also examined stimulus-preceding negativity (SPN; ref. 37) after the presentation of the response information but before the presentation of the feedback information. Such slow wave activity is thought to represent a variety of processes including sensitivity to forthcoming outcome and affective information^38^ and as such may contribute to FRN modulation further downstream.

## Results

### Behavioural data

#### Trial n item selection and outcome

In terms of the proportion of trials distributed across item and outcome, participants did not differ in their item selection at trial *n* [F(2, 70) = 2.09, MSE = 0.013, *p* = 0.132, η_p_^2^ = 0.056] nor did this differ according to condition [F(4, 140) = 1.64, MSE = 0.002, p = 0.167, η_p_^2^ = 0.045]. There was a numerical tendency to play Rock slightly more often than Paper or Scissors (35.16%, 32.50% and 32.35% trials, respectively), consistent with previous studies^7^,^8^,^13^. There was no significant effect of outcome at trial *n* [F(2, 70) = 0.02, MSE = 0.002, *p* = 0.982, η_p_^2^ < 0.001] and no interaction with condition [F(4, 140) = 1.58, MSE = 0.002, *p* = 0.182, η_p_^2^ = 0.043]. Winning, losing and drawing were all around 33.3% (33.38%, 33.26% and 33.36% trials, respectively), as would be expected from playing an opponent adopting the mixed-equilibrium strategy.

#### First-order repetition effects

Proportion data using last 149 trials in each condition (the first trial in each block had no previous history) were analysed according to a three-way repeated measures ANOVA using the factors of condition (*baseline*, *win-heavy*, *lose-heavy*), outcome at trial *n* (*win*, *lose*, *draw*) and strategy at trial *n* + *1* (*stay*, *upgrade*, *downgrade*; after ref. 7). The main effect of strategy was not significant [F(2, 70) = 1.04, MSE = 0.140, *p* = 0.359, η_p_^2^ = 0.029], nor was the two-way interaction between condition × strategy at trial *n* + *1* [F(4, 140) = 2.24, MSE = 0.022, *p* = 0.068, η_p_^2^ = 0.060]. However, a significant two-way interaction between outcome × strategy at trial *n* + *1* [F(4, 140) = 18.17, MSE = 0.051, *p* < 0.001, η_p_^2^ = 0.342] was observed. This showed a larger proportion of *stay* trials relative to the two forms of switching, *upgrading* or *downgrading*, following a *win* (42.45%, 29.32% and 28.23%, respectively), thereby providing support for the *win-stay* heuristic. The *lose-shift* heuristic was also in evidence by a larger proportion of *upgrading* and *downgrading* (numerically in favour of *downgrading*) relative to *staying* following a *loss* (39.13%, 39.41% and 21.46%, respectively). Finally, *upgrading* was numerically more popular than both the other form of switch- *downgrading*- and *staying* during *draw* trials, although the differences between these forms of strategy (37.32%, 33.40% and 29.28%, respectively) were not statistically significant. The relationships between *win-stay*, *lose-downgrade* and *draw-upgrade* replicate the main findings of^7^ and show broad equivalence in performance between Canadian and UK samples.

**Figure 1:**
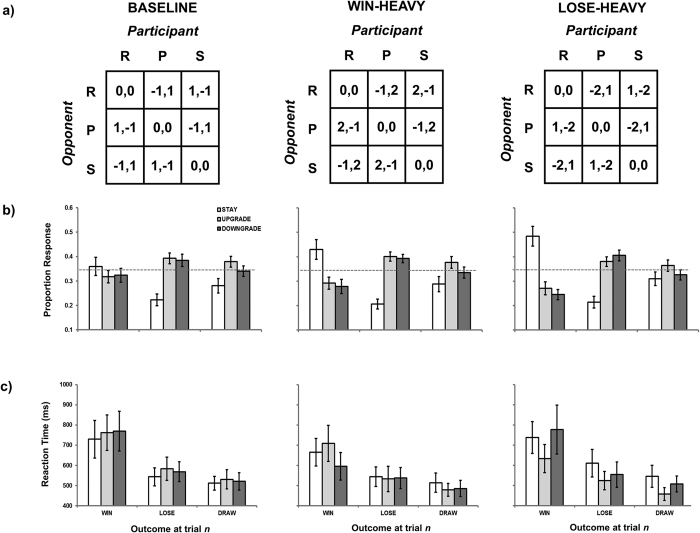
(**a**) Pay-off matrices for three *baseline*, *win-heavy* and *lose-heavy* conditions (R = Rock, P = Paper, S = Scissors). (**b**) Proportion response and (**c**) reaction time data as a function of condition, outcome at trial *n* and strategy at trial *n* + *1*.

A significant three-way interaction [F(8, 280) = 2.98, MSE = 0.012, *p* = 0.003, η_p_^2^ = 0.079; see Fig. 1b] was relatively straightforward in its interpretation in that the distribution of *stay*, *upgrade* and *downgrade* responses remained stable across *lose* and *draw* trials, but the proportion of *stay* responses following *win* trials was significantly larger during *win-heavy* conditions relative to the *baseline* condition (48.44% versus 35.92%, respectively; Tukey’s HSD, *p* < 0.05). *Win-stay* responses were also increased during *lose-heavy* condition (42.99%) but this proportion was not statistically significant from the *baseline* condition (Tukey’s HSD, *p* > 0.05). Modulating the value of wins and losses increased the likelihood of the *win-stay* heuristic, but did not change the frequency of the *lose-shift* heuristic. These data suggest that participants retain more cognitive control over decision-making following a *win* (e.g., System 2) and retain less cognitive control over decision making following a *loss* or a *draw* (e.g., System 1).

#### Reaction time

To further test the idea that relatively controlled decisions follow positive outcome (*win*) but relatively automatic decisions follow negative outcome (*loss* or *draw*), median RT was calculated per participant for each of the nine possible outcome-strategy combinations for all three conditions. Four participants had to be rejected as a result of failing to have any observations in some of these 27 cells, and a further three participants were rejected as a result of their average median RT (2116, 2068.5 and 1476.5 ms) being at least twice as large as the group average median RT (583 ms). Data from the remaining 29 participants were entered into a three-way repeated measures ANOVA using the factors of condition (*baseline*, *win-heavy*, *lose-heavy*), outcome at trial *n* (*win*, *lose*, *draw*) and strategy at trial *n* + *1* (*stay*, *upgrade*, *downgrade;* see Fig. 1c). Main effects of condition, strategy, and interactions between condition × outcome, outcome × strategy, and, condition × outcome × strategy all failed to reach statistical significance (*F* < 1). An interaction between condition × strategy [F(4, 100) = 2.82, MSE = 42529, *p* = 0.029, η_p_^2^ = 0.091] failed to show any pairwise statistical differences using Tukey’s HSD (*p* < 0.05; c.f.,^39^, *non- consonance*). However, the main effect of outcome [F(2, 56) = 17.99, MSE = 162685, *p* < 0.001, η_p_^2^ = 0.391] revealed that responses following wins were slower than responses following losses or draws (708, 555 and 505 ms, respectively; Tukey’s HSD, *p* < 0.05). Slower responses are consistent with the increase in cognitive control participants are able to exert following *win* trials relative to *lose* or *draw* trials.

#### ERP data

**Figure 2:**
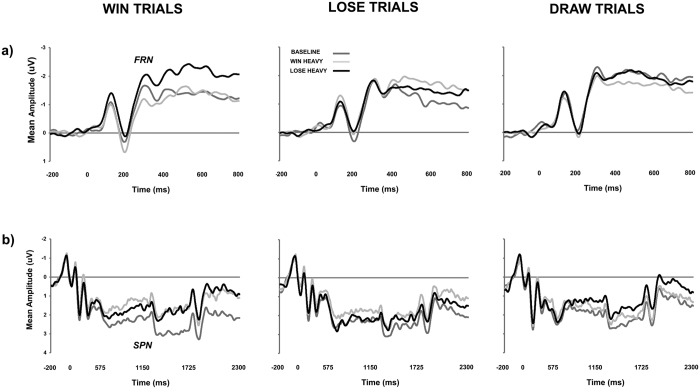
(**a**) Group-average ERP generated by trial outcome (FRN = feedback-related negativity). (**b**) Group-average ERP generated by response presentation (SPN = stimulus-preceding negativity). ERPs are collapsed across nine fronto-central electrodes (F1, Fz, F2, FC1, FCz, FCz, C1, Cz, C2). The data are filtered at 20 Hz for graphic illustration only.

**Figure 3:**
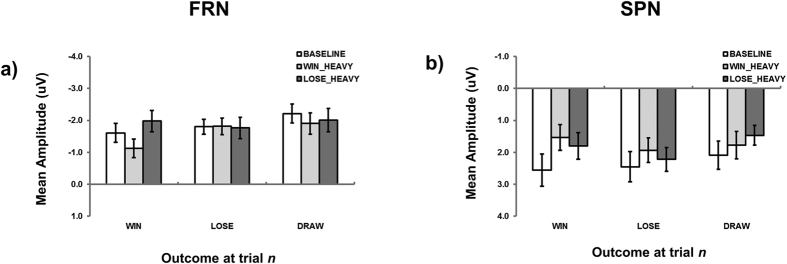
(**a**) FRN and (**b**) SPN mean amplitude as a function of condition and outcome at trial *n*. Error bars represent ±1standard error.

FRN mean amplitude was compared across the three conditions (*baseline*, *win-heavy*, *lose-heavy*) for each of the three trial outcomes (*win*, *lose*, *draw*; see Fig. 2a) within a two-way repeated measures ANOVA. The main effect of outcome was significant [F(2, 70) = 3.34, MSE = 1.806, *p* = 0.041, η_p_^2^ = 0.087], revealing that *draw* trials generated significantly greater FRN than *win* trials (−1.57 versus −2.04 μV; Tukey’s HSD; *p* < 0.05). This further underscores the importance of considering *draw* trials as an additional example of negative outcome. The main effect of condition [F(2, 70) = 1.52, MSE = 1.927, *p* = 0.225, η_p_^2^ = 0.042] and interaction failed to reach statistical significance [F(4, 140) = 2.37, MSE = 0.970, *p* = 0.055, η_p_^2^ = 0.063]. On the basis of the principle of *incoherence*^39^ where omnibus statistics from ANOVA may fail to reveal specific pairwise comparisons, and predicated on the basis of previous research^35^ and our own behavioural data featuring the modulation of performance following *win* but not *lose* or *draw* trials, FRN mean amplitude was compared across the three conditions (*baseline*, *win-heavy*, *lose-heavy*) separately for each of the three trial outcomes (*win*, *lose*, *draw*). This revealed a main effect of condition during *win* trials [F(2, 70) = 5.07, MSE = 3.907, *p* = 0.009, η_p_^2^ = 0.127], but not for *lose* [F(2, 70) = 0.02, MSE = 3.244, *p* = 0.978, η_p_^2^ < 0.001] or *draw* [F(2, 70) = 0.61, MSE = 4.448, *p* = 0.545, η_p_^2^ = 0.017] trials. The modulation of FRN was only apparent following *win* feedback, with larger negativity being generated for *lose-heavy* relative to *win-heavy* conditions (Tukey’s HSD; *p* < 0.05, see Fig. 3a).

A similar two-way repeated measures ANOVA on SPN mean amplitude (see Fig. 2b) failed to reveal main effects of condition [F(2, 70) = 2.35, MSE = 5.205, *p* = 0.109, η_p_^2^ = 0.063] or outcome [F(2, 70) = 2.33, MSE = 2.107, *p* = 0.105, η_p_^2^ = 0.062], or, a significant interaction between condition × outcome [F(2, 70) = 1.53, MSE = 1.268, *p* = 0.197, η_p_^2^ = 0.041]. However as per FRN, with a separate consideration of each of the three potential outcomes, a main effect of condition was revealed during *win* trials [F(2, 70) = 3.72, MSE = 2.740, *p* = 0.029, η_p_^2^ = 0.097], but not for *lose* [F(2, 70) = 0.89, MSE = 2.747, *p* = 0.417, η_p_^2^ = 0.025] or *draw* [F(2, 70) = 1.54, MSE = 2.254, *p* = 0.221, η_p_^2^ = 0.004] trials. The modulation of SPN was only apparent following *win* trials, with larger negativity being generated for *win-heavy* and *lose-heavy* relative to *baseline* conditions (Tukey’s HSD; *p* < 0.05, see Fig. 3b).

## Discussion

In contrast to previous research that demonstrate the use of relatively fast, automatic and emotional (System 1) versus more slow, controllable and reasoned (System 2) processing^3^ across separate tasks^40^, we show that such distinctions are available within the same game space (RPS) and in part determined by the outcome of the previous trial. Critically, we note the *win-stay lose-shift* heuristic is not a unified mechanism. Specifically, the relative speed with which responses following losing are initiated, the inflexibility in the magnitude of *lose-shift* strategy, and, the lack of significant neural FRN or SPN modulation within *lose* trials as a function of outcome value, point towards *lose-shift* behaviour relying heavily on System 1 processes. In contrast, the relatively slow responding following *win* trials, the flexibility in the magnitude of *win-stay* play, and, the modulation of FRN and SPN amplitude in *win* trials [see also^35^] following changes in outcome value, point towards *win-stay* behaviour relying heavily on System 2 processes.

We also found that this potential haven of System 2 operations following *win* outcomes in a *baseline* condition (see also^7^), wherein participants equally (and more slowly; ref. 41) distribute their responses across the three potential strategies, was compromised by manipulating outcome value. That is, making win trials more valuable (either by attempting to equate their subjective value with losses: *win-heavy*; or, making losses particularly damaging: *lose-heavy*) increased the likelihood of deploying a *win-stay* strategy, thereby increasing behavioural predictability and potentially compromising successful performance in a competitive environment. This increase in irrationality following a win in conditions where outcome value was manipulated was also marked by increases in FRN and SPN. These data are in alignment with the contention FRN may be sensitive to the magnitude of trial outcomes when that outcome is positive (*win*; ref. 35) rather than negative (*lose*; ref. 34). This provides further evidence that the processes evoked following the experience of a *win* are quite different from the processes evoked following the experience of a *loss*.

This is not to deny that *win-stay* mechanisms will always be more flexible than *lose-shift* mechanisms. For example, in a recent investigation of outcome value^6^, a win trial was set at a variable value of *a* (typically 2 and above) whereas draw trials were always assigned a value of 1 and loss trials a value of 0. It was found that as the variable value of *a* (incentive) increased, so too did the frequency of *lose-downgrade* responses (‘lose-left-shift’, in their terminology) but the frequency of *win-stay* responses actually decreased. Here, there appears to be contrast with the data from our *win-heavy* condition (where win = +2 and loss = −1 and so ‘incentive’ was also high) in which the frequency of *win-stay* responses actually increased relative to *baseline* condition, while the preponderance of *lose-downgrade* trials remained largely unchanged. One potential resolution may be found in comparing the differing ways in which the various payoff matrices have been conceptualized. There are two issues here: First, we implemented RPS under minimal reward conditions in that points accrued during the game were not converted into money at the end of the experimental session. Therefore, there may be broad differences in the perceived value of outcome when task performance is (and is not) linked to financial inventive^7^. Second, *loss* trials as described in^6^ do not have the negative valence traditionally associated with them but rather are defined by the absence of positive value, and *draw* trials are actually ascribed a positive value despite both behavioural and neural evidence (refs 6, 29, 42) suggesting that a tie with an opponent represents a negative rather than positive trial outcome. Therefore, although the relative differences between *wins*, *losses* and *draws* are identical between^6^ (where *a* = 2) and the current *baseline* condition, outcomes in the former case do not appear to have the same valence as outcomes in the latter case (2, 1, 0 versus +1, 0, −1, respectively). The framing of wins and losses is a well-established literature (see ref. 43 for a review) and it appears germane for future research to resolve these potential differences, especially given the potential difference between objective and subjective outcome values explored here. This is of course in addition to acknowledging that while we have focused on a group aggregate of performance here, there exists significant variation in individual strategy across games^44^.

In sum, our ability to maintain rational decisions in competitive environments appears limited to winning and only when the value of wins are low. The observation that we reliably perpetuate poor decisions following a loss^4^,^7^ and now also, following high-yield wins, pulls into focus the possible avenues for behavioural modification in problem gamblers or individuals predisposed to developing addictions, and highlights how vulnerable we can make ourselves when attempting to strategize in a competitive environment.

### Materials

#### Participants

36 undergraduate students (30 female) participated in the study; mean age was 21.22 years (SD = 3.98) and all were right-handed. Three additional participants were removed due to noisy EEG data. The study was approved for testing by the Life Sciences and Psychology Research Ethics Committee (C-REC) at the University of Sussex (ER/BJD21/3), and the study was carried out in accordance with the approved guidelines. Informed consent was obtained from all participants, and all individuals received either course credit or £20 for participation. Compensation was independent of task performance.

#### Stimuli and apparatus

Composite pictures of two interacting hands making Rock, Paper and Scissors signs were displayed at a visual angle of approximately 12° × 6° with participants sat approximately 57 cm away from the screen. The presentation of stimuli was controlled by Presentation 18.1 (build 03.31.15; neurobs.com) and responses were recorded using a keyboard. Participants wore a white glove during experimentation.

#### Design and procedure

Participants completed 450 trials of RPS separated across 3 counterbalanced blocks (*baseline*, *win-heavy*, *lose-heavy*) of 150 trials each. At the bottom of the screen, the cumulative scores for both computer (on the left) and player (on the right) were displayed, in addition to the trial count within that block. In each block, the computer played Rock, Paper and Scissors 50 times in a random order. At each trial, participants were prompted to press one of three buttons corresponding to Rock, Paper and Scissors, promoted by the presentation of a fixation cross. At the time of pressing, the computer displayed a composite picture showing the result of the RPS trial, with its selection on the left (depicted by a blue glove) and the participant’s selection on the right (depicted by a white glove) for 1000 ms. Following the clearing of the picture (500 ms), feedback was provided for a further 1000 ms centre screen as to whether the participant won, lost or drew the trial. Scores were then updated during a 500 ms period according to the criteria set out in Fig. 1a and the next trial began with a fixation cross. Participants were informed that the computer would play in a certain way (which would be revealed after the experiment) and that they were to try to beat the computer across the course of the game.

#### Questionnaire administration

To maintain parity with other RPS experiments in the lab, three short questionnaires were administered following the completion of each RPS block to assess individual’s degree of engagement with the block, the degree of anthropomorphism assigned to the computerized opponent, and co-presence felt between the player and opponent. First, engagement with specific blocks of RPS was assessed using a slightly-modified Game Engagement Questionnaire (GEQ; ref. 45). Eighteen from 19 original items were evaluated on a 5-point scale, encompassing the factors of *absorption*, *flow*, *presence* and *immersion*. The one item to be dropped from the revised GEQ was ‘I play longer than I meant to’ (related to *presence*). This item was deemed inappropriate given the fixed number of rounds in all RPS block. All items were also re-written to refer to the past tense (e.g., ‘I lose track of time’ became ‘I lost track of time’). Second, the degree of anthropomorphism attributed to the computer opponent was assessed on the basis of^46^ (Study 1) where five anthropomorphic states (‘mind of its own’, ‘intentions’, ‘free will’, ‘consciousness’, ‘experienced emotion’) and three non-anthropomorphic states (‘attractive’, ‘efficient’, ‘strong’) were adjudicated on an 11-point scale (c.f.^47^, Box A1). Third, aspects of self-reported co-presence and perceived other’s co-presence were adapted from the scales provided by^48^. Seven modified items, evaluated on a 5-point scale, were deemed appropriate for opponent interaction in the context of RPS (three related to self-reported co-presence, and four related to perceived other’s co-presence; see Appendix). A further five unique items were added to this particular questionnaire to assess the participant’s understanding of the opponent’s strategy: “I felt as though my opponent had a strategy that was based on the moves I was making” (e.g., *other* strategy), “I felt as though my opponent had a strategy that was based on the moves it was making” (e.g., *self* strategy), “My opponent exhibited a human-like strategy”, “I felt like my opponent was somehow cheating”, and “I found this block of RPS rewarding to play.” Finally, to assess the empathy of the participant, the Toronto Empathy Questionniare (TEQ; ref. 49) was administered after all blocks of RSP have been played. The TEQ consists of 16-items judged on a 5-point scale, yielding a single empathy factor.

#### ERP recording

Electrical brain activity was continuously digitized using a 64 channel ANT Neuro amplifier and a 1000 Hz sampling rate. Horizontal and vertical eye movements were also recorded using channels placed at the outer canthi and at inferior orbits, respectively. Data processing was conducted using BESA 5.3 Research (MEGIS; Gräfelfing, Germany). The contributions of both vertical and horizontal eye movements were reduced from the EEG record using the VEOG and HEOG artefact options in BESA following average referencing. Using a 0.1 Hz (12 db/oct; zero phase) high-pass and 30 Hz (24 db/oct; zero phase) low-pass filter, epochs were defined relative to the onset of the feedback (to assess FRN) and response presentation (to assess SPN). Epochs were baseline corrected according to a 200 ms pre-stimulus interval and neural activity was examined 800 ms post-feedback in the case of FRN and 2300 ms post-response presentation in the case of SPN. FRN mean amplitude was calculated on the basis of a 50 ms window centered around the peak latency (between 225 – 350 ms) reported for each specific condition and according to the anterior-posterior position on the scalp (F1, Fz, F2; FC1, FCz, FC2; C1, Cz, C2). SPN mean amplitude was calculated between 600 and 1600 ms from the same nine fronto-central electrodes. Individual epochs were rejected on the basis of amplitude difference exceeding 100 μV, gradient between consecutive time points exceeding 75 μV, or, signal lower than 0.01 μV, within any channel.

## Additional Information

**How to cite this article**: Forder, L. and Dyson, B. J. Behavioural and neural modulation of *win-stay* but not *lose-shift* strategies as a function of outcome value in Rock, Paper, Scissors. *Sci. Rep.* **6**, 33809; doi: 10.1038/srep33809 (2016).

## References

[b1] Decety J., Jackson P. L., Sommerville J. A., Chaminade T. & Meltzoff A. N.. The neural basis of cooperation and competition. NeuroImage 23, 744–751 (2004). 15488424 10.1016/j.neuroimage.2004.05.025 PMC3640982

[b2] Zeelenberg M. & Beattie J.. Consequences of regret aversion 2: Additional evidence for effects of feedback on decision making. Organ Behav Hum Dec 72, 63–78 (1997).

[b3] Kahneman D.. Thinking, Fast and Slow (Farrar, Straus and Giroux, New York, 2011).

[b4] Laakasuo M., Palomäki J. & Salmela J. M.. Emotional and social factors influence poker decision making accuracy. J Gambl Stud 31, 933–947 (2015). 24633674 10.1007/s10899-014-9454-5

[b5] Wan X. . The neural basis of intuitive best next-move generation in board game experts. Science 331, 341–346 (2011). 21252348 10.1126/science.1194732

[b6] Wang Z. & Xu B B.. Incentive and stability in the Rock-Paper-Scissors game: An experimental investigation. e-print rXiv/1407. 1170 (2014).

[b7] Dyson B. J., Wilbiks J. M. P., Sandhu R., Papanicolaou G. & Lintag J.. Negative outcomes evoke cyclic irrational decisions in Rock, Paper, Scissors. Sci Reps 6, 20479 (2016). 10.1038/srep20479 PMC4740902 26843423

[b8] Baek K. . Response randomization of one- and two-person Rock-Paper-Scissors games in individuals with schizophrenia. Psychiat Res 207, 158–163 (2013). 10.1016/j.psychres.2012.09.003 23017652

[b9] Zhou. H.-J. The rock-paper-scissors game. Contem Phys 10.1080/00107514.2015.1026556 (2015).

[b10] Abe H. & D. Lee H.. Distributed coding of actual and hypothetical outcomes in the orbital and dorsolateral prefrontal cortex. Neuron 70, 731–741 (2011). 21609828 10.1016/j.neuron.2011.03.026 PMC3104017

[b11] Bi Z. & Zhou H.-J.. Optimal cooperation trap strategies for the iterated rock-paper-scissors game. PLoS One 9, e111278 (2014). 25354212 10.1371/journal.pone.0111278 PMC4213018

[b12] Lee D., McGreevy B. P. & Barraclough D. J.. Learning and decision making in monkeys during a rock-paper-scissors game. Cognitive Brain Res 25, 416–430 (2005). 10.1016/j.cogbrainres.2005.07.003 16095886

[b13] Wang Z., Xu B. & Zhou H-J.. Social cycling and conditional responses in the Rock-Paper-Scissors game. Sci Reps 4, 5830 (2014). 10.1038/srep05830 PMC5376050 25060115

[b14] Thorndike E. L.. Animal Intelligence (Macmillan Company, New York, 1911).

[b15] Toupo D. F. P. & Strogatz S. H. Nonlinear dynamics of the rock-paper-scissors game with mutations. Phy Rev E91, 052907 (2015). 10.1103/PhysRevE.91.052907 26066229

[b16] Rayburn-Reeves R. M., Molet M. & Zentall T. R.. Simultaneous discrimination reversal learning in pigeons and humans: Anticipatory and perseverative errors. Learn Behav 39, 125–137 (2011). 21264566 10.3758/s13420-010-0011-5

[b17] Olton D. S. & Schlosberg P.. Food-searching strategies in young rats: Win-shift predominates over win-stay. J Comp Physiol Psychol 92, 609–618 (1978).

[b18] Bolles R. C.. Species-specific defense reactions and avoidance learning. Psychol Rev 77, 32–48 (1970).

[b19] Thaler R. H., Tversky A., Kahneman D. & Schwartz A.. The effect of myopia and loss aversion on risk taking: An experimental test. Q J Econ 112 647–661 (1997).

[b20] Sun Z. . Attentional bias in competitive situations: Winner does not take all. Front Psychol 6, 1469 (2015). 26441814 10.3389/fpsyg.2015.01469 PMC4585104

[b21] Gigerenzer G. & Goldstein D. G.. Reasoning the fast and frugal way: Models of bounded rationality. Psychol Rev 103, 650–669. 8888650 10.1037/0033-295x.103.4.650

[b22] Miltner W. H. R., Braun C. H. & Coles M. G. H.. Event related brain potentials following incorrect feedback in a time estimation task: Evidence for a generic neural system for error detection. J Cognitive Neurosci 9, 787–796 (1997). 10.1162/jocn.1997.9.6.788 23964600

[b23] Hauser T. U. . The feedback-related negativity (FRN) revisited: New insights into the localization, meaning and network organization. NeuroImage 84, 159–168 (2014). 23973408 10.1016/j.neuroimage.2013.08.028

[b24] Holroyd C. B. & Krigolson O. E.. Reward prediction error signals associated with a modified time estimation task. Psychophysiology 44, 913–917 (2007). 17640267 10.1111/j.1469-8986.2007.00561.x

[b25] Gehring W. J. & Willoughby A. R.. The medial frontal cortex and the rapid processing of monetary gains and losses. Science 295, 2279–2282 (2002). 11910116 10.1126/science.1066893

[b26] Holroyd C. B., Larsen J. T. & Cohen J. D.. Context dependence of the event-related potential associated with reward and punishment. Psychophysiology 41, 245–253 (2004). 15032989 10.1111/j.1469-8986.2004.00152.x

[b27] Gentsch A., Ullsperger P. & Ullsperger M.. Dissociable medial frontal negativities from a common monitoring system for self- and externally caused failure of goal achievement. NeuroImage 47, 2023–2030 (2009). 19486945 10.1016/j.neuroimage.2009.05.064

[b28] Frank M. J., Woroch B. S. & Curran T.. Error-related negativity predicts reinforcement learning and conflict biases. Neuron 47, 495–501 (2006). 16102533 10.1016/j.neuron.2005.06.020

[b29] Holroyd C. B., Hajcak G. & Larsen J. T.. The good, the bad and the neutral: Electrophysiological responses to feedback stimuli. Brain Res 1105, 93–101 (2006). 16427615 10.1016/j.brainres.2005.12.015

[b30] Luft C. D. B.. Learning from feedback: The neural mechanisms of feedback processing facilitating better performance. Behav Brain Res 261, 356–368 (2014). 24406725 10.1016/j.bbr.2013.12.043

[b31] Müller S. V., Möller J., Rodriguez-Fornells A. & Münte T. F.. Brain potentials related to self-generated and external information used for performance monitoring. ClinNeurophysiol 116, 63–741 (2006). 10.1016/j.clinph.2004.07.009 15589185

[b32] Heldmann M., Russeler J. & Münte T. F.. Internal and external information in error processing. BMC Neurosci 9, 33 (2008). 18366727 10.1186/1471-2202-9-33 PMC2291472

[b33] Yeung N. & Sanfey A. G.. Independent coding of reward magnitude and valence in the human brain. J Neurosci 24, 6258–6264 (2004). 15254080 10.1523/JNEUROSCI.4537-03.2004 PMC6729539

[b34] Hajcak G., Moser J. S., Holroyd C. B. & Simons. R. F. The feedback-related negativity reflects the binary evaluation of good versus bad outcomes. Biol Psychol 71, 148–154 (2006). 16005561 10.1016/j.biopsycho.2005.04.001

[b35] Cohen M. X., Elger C. E. & Ranganath C.. Reward expectation modulates feedback-related negativity and EEG spectra. Neuroimage 35, 968–978 (2007). 17257860 10.1016/j.neuroimage.2006.11.056 PMC1868547

[b36] Baker T. E. & Holroyd C. B.. Which way do I go? Neural activation in response to feedback and spatial processing in a virtual T-Maze. Cereb Cortex 19, 1708–1722 (2009). 19073622 10.1093/cercor/bhn223

[b37] Donkers F. C. L. & van Boxtel G. J. M.. Mediofrontal negativities to averted gains and losses in the slot-machine task: A further investigation. J Psychophysiol 19, 256–262 (2005).

[b38] van Boxtel G. J. M. & Böcker K. B. E.. Cortical measures of anticipation. J Psychophysiol 18, 61–76 (2004).

[b39] Gabriel K. R.. Simultaneous test procedures- some theory of multiple comparisons. Ann Math Stat 40, 224–250 (1969).

[b40] Kuo W.-J., Sjostrom T., Chen Y.-P., Wang Y.-H. & Huang C.-Y. Intuition and deliberation: Two systems for strategizing in the brain. Science 324, 519–522 (2009). 19390048 10.1126/science.1165598

[b41] Herz D. M., Zavala B. A., Bogacz R. & Brown P.. Neural correlates of decision thresholds in the human subthalamic nucleus. Curr Bio 26, 1–5 (2016). 26996501 10.1016/j.cub.2016.01.051 PMC4826434

[b42] Vickery T. J., Chun M. M. & Lee D.. Ubiquity and specificity of reinforcement signals throughout the human brain. Neuron 72, 166–177 (2011). 21982377 10.1016/j.neuron.2011.08.011

[b43] Tversky A. & Kahneman D.. The framing of decisions and the psychology of choice. Science 211, 453–458 (1981). 7455683 10.1126/science.7455683

[b44] Worthy D. A., Hawthorne M. J. & Otto A. R.. Heterogenity of strategy use in the Iowa gambling task: a comparison of win-stay/lose-shift and reinforcement learning models. Psychon B Rev 20, 364–371 (2013). 10.3758/s13423-012-0324-9 23065763

[b45] Brockmeyer J. H. . The development of the Game Engagement Questionnaire: A measure of engagement in video game-playing. J Exp Soc Psychol 45, 624–634 (2009).

[b46] Epley N., Akalis S., Waytz A. & Cacioppo J. T.. Creating social conncetion through inferential reproduction: Loneliness and perceived agency in gadgets, gods, and greyhounds. Psychol Sci 19, 114–120 (2008). 18271858 10.1111/j.1467-9280.2008.02056.x

[b47] Waytz A., Cacioppo J. T. & Epley N.. Social cognition unbound: Insights into anthropomorphism and dehumanization. Curr Dir Psychol Sc 19, 58–62 (2010). 24839358 10.1177/0963721409359302 PMC4020342

[b48] Nowak K. L. & Biocca F.. The effect of the agency and anthropomorphism on users’ sense of telepresence, copresence, and social presence in virtual environments. Presence-Teleop Virt 12, 481–494 (2003).

[b49] Spreng N., McKinnon, Mar M. & Levine B.. The Toronto empathy questionnaire: Scale development and initial validation of a factor-analytic solution to multiple empathy measures. J Pers Asssess 91, 62–71 (2009). 10.1080/00223890802484381 PMC2775495 19085285

